# Corneal endothelial changes seven years after phacoemulsification cataract surgery

**DOI:** 10.1007/s10792-024-03044-6

**Published:** 2024-04-08

**Authors:** Björn Lundberg

**Affiliations:** Department of Clinical Science/Ophthalmology, Umeå University, 901 85 Umeå, Sweden

**Keywords:** Corneal endothelial cell loss, Corneal endothelial cell count, Corneal oedema, Long-term corneal endothelial changes

## Abstract

**Purpose:**

To evaluate long-term postoperative corneal changes after phacoemulsification cataract surgery.

**Methods:**

Twenty patients who participated in a previous study regarding corneal endothelial changes after phacoemulsification cataract surgery were examined after 7 years. The patients were divided in three groups based on their initial increase in central corneal thickness day one after the surgery: < 5% increase, 6–20% increase and ≥ 20% increase. The primary outcome measures were corneal endothelial cell loss (ECL), endothelial cell count (ECC) and endothelial morphology.

**Results:**

After 7 years, a difference in cell loss between the groups was observed, except for groups 1 and 2. Endothelial cell count (ECC) differed significantly between groups 1 and 3 at 3 months. At 7 years, there was no difference in ECC between the three groups. Cell loss was found exclusively in group 1 between 3 months and 7 years. Endothelial cell morphology showed a converging pattern between 3 months and 7 years.

**Conclusion:**

After phacoemulsification cataract surgery, long-term ECC and morphology appear to converge towards a comparable steady state regardless of initial corneal swelling and endothelial cell loss.

## Introduction

Corneal endothelial cell loss (ECL) after phacoemulsification cataract surgery is a thoroughly studied postoperative parameter and is an important variable, not least because it is associated with the long-term risk for corneal decompensation (pseudophakic bullous keratopathy).[1] The ECL is caused by the surgical trauma, i.e. the corneal incision, irrigation fluid and effects of the ultrasound. Despite some contradictory results, several intraoperative parameters have been pointed out to influence the ECL after cataract surgery—axial length, length of corneal incision, phacoemulsification time and energy, anterior chamber depth, posterior capsule rupture and nucleus grade.[2–5] In a previous study, we found that nucleus colour was the only independent variable that was correlated to central ECL [6].

It is affirmed that there is a progressive ECL after cataract surgery that exceeds the physiological cell loss. Studies concerning endothelial cell loss (ECL) predominantly have follow-up times of a year or less and there is a wide range in reported ECL after cataract surgery, i.e. 2.3–16.6% [2–14.] A few studies have follow-up times up to 1–3 years, but it is still uncertain what happens in an even longer period—is the cell loss progressive or does it level off?

Apart from the ECL, corneal endothelial morphology changes after cataract surgery may reflect a more functional aspect that is not directly correlated to the ECL. After the surgical trauma, the endothelial cells become more irregular regarding size, hexagonality and shape. This is reflected by the parameters coefficient of variation (CV), hexagon shape factor (HSF) and degree of elongation (DE). Changes in these parameters have been shown to stabilize over time after cataract surgery, indicating that the corneal endothelial cells have come to rest [2, 6].

The aim of the present study was to examine how endothelial cell parameters appeared after phacoemulsification cataract surgery in a long-term perspective.

## Methods

Prospective observational case series, in accordance with the Declaration of Helsinki and approved by the research ethics committee of Umeå University, Umeå, Sweden (UM dnr 04-033M). Three groups of patients were analysed according to the classification in the previous study—the subjects were divided into the groups based on their initial increase in central corneal thickness at day one after the cataract procedure—group 1: < 5% increase, group 2: 6–20% increase and group 3: ≥ 20% increase.

An additional classification was analysed based on if the corneas were defined as “clear” and “not clear” depending on if a specular microscope endothelial photography at day one could be obtained or not [6].

Thirty patients from the previous study were contacted 7 years after the cataract surgery. The operated eye from each patient in the original study was followed up. Nine patients had died, and one patient declined participation. One patient was excluded since the measurements were not accurate due to lack of cooperation. Groups 1, 2 and 3 included six, six and seven patients, respectively.

Orbscan II (Bausch & Lomb Surgical, San Dimas, California, USA) anterior segment slit-scan tomography was performed for corneal thickness. Corneal endothelial photographs were taken with the Topcon SP-2000P specular microscope (Topcon Europe B.V., Capelle a/d Ijssel, The Netherlands). Both devices were the same as used in the first study. Corneal endothelial morphology was calculated from a cluster of 55 cells from each photograph as previously detailed[6.] The endothelial cell count, the hexagon shape factor (HSF) (quantifying the deviation from the ideal hexagonal cell shape), the degree of cell elongation (DE) and the coefficient of variation in cell size (CV) were calculated.

Best-corrected visual acuity (BCVA) was measured using the Early Treatment of Diabetic Retinopathy Study fast protocol, and intraocular pressure (IOP) was measured with Goldmann applanation tonometry.

## Statistical analysis

All statistical analyses were performed with SPSS software (IBM, version 27). The Mann–Whitney U-test was used to test for two independent samples. The Wilcoxon signed-rank test was used for two related samples. A *P* value < 0.05 was considered statistically significant.

## Results

There was a significant difference in age between groups 1 and 2/3, where group 1 had younger patients. There also was a difference between the groups with corneas defined as “clear” vs. “not clear”, with younger patients in the former.

No significant differences between the three groups or between clear and not clear corneas were observed preoperatively regarding any other parameter—visual acuity, intraocular pressure, corneal thickness, endothelial cell count (ECC) and morphology (Table 1). Twelve corneas were defined as clear and seven as not clear.

**Table 1:** Selected parameters presurgical and after 7 years

Group^a^	VA^b^pre	CCT^c^pre	ECC^d^pre	VA7y^e^	CCT7y	ECC7y	Age7y
1	0.60 ± 0.33	563 ± 32	2898 ± 472	−0.02 ± 0.07	558 ± 36	2442 ± 512	73 ± 6
2	0.45 ± 0.15	541 ± 24	2763 ± 390	0.14 ± 0.21	533 ± 16	2182 ± 416	86 ± 2
3	0.46 ± 0.25	576 ± 41	3228 ± 443	0.17 ± 0.25	566 ± 36	1989 ± 368	85 ± 4
Clear	0.52 ± 0.26	552 ± 30	2830 ± 419	0.06 ± 0.17	545 ± 29	2312 ± 470	80 ± 8
Not clear	0.54 ± 0.25	567 ± 41	3228 ± 443	0.18 ± 0.25	558 ± 36	1989 ± 368	86 ± 4

^a^Increase in central corneal thickness day one after the cataract surgery—group 1: < 5% increase, group 2: 6% to 20% increase, and group 3: ≥ 20% increase. Corneas were defined as “clear” and “not clear” depending on if a specular microscope endothelial photography at day one could be obtained or not

^b^VA: Visual acuity (ETDRS)

^c^CCT: Central corneal thickness (μm)

^d^ECC: Endothelial cell count (1/mm^2^)

^e^Years

At 3 months, a difference in ECL between groups 1 and 3, and 2 and 3 was shown, with a greater loss in group 3. There was no significant difference in ECL at 3 months between groups 1 and 2*.* The ECL between 3 months and 7 years was significantly different between groups 1 and 3 but not for the other groups. The cell loss was greater in group 1 in this interval. At 7 years, compared to preoperative numbers, ECL was not significant between groups 1 and 2, but significant between the other groups.

At 7 years, there was a significant ECL within all groups. At 3 months, there was no ECL within group 1, but within groups 2 and 3 a significant loss was observed. The opposite was shown between 3 months and 7 years—a significant ECL was found only within group 1 but not within groups 2 and 3.

**Fig. 1:**
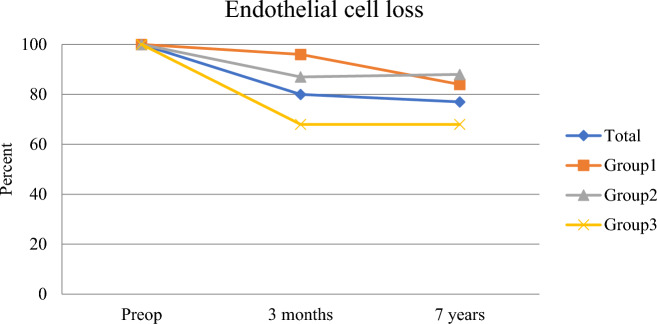
At 3 months: significant difference between group 1 and 3, 2 and 3, with a greater loss in group 3. Non significant difference between group 1 and 2. Between 3 months and 7 years: significant difference between group 1 and 3 but not for the other groups. The cell loss was greater in group 1 in this interval. Between preop and 7 years: non significant between group 1 and 2, but significant between the other groups. At 7 years: significant ECL within all groups. At 3 months: non significant ECL within group 1 but significant within group 2 and 3. The opposite was seen between 3 months and 7 years - a significant ECL came out only within group 1 but not within group 2 and 3. ECL: Endothelial cell loss preoperatively, at 3 months and 7 years. Increase in central corneal thickness day one after the cataract surgery—group 1: < 5% increase, group 2: 6% to 20% increase, and group 3: ≥ 20% increase. Note the converging pattern up to 7 years

**Fig. 2:**
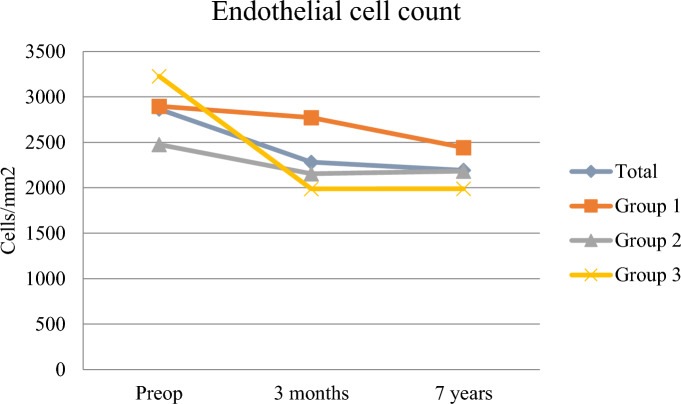
There is a significant difference in ECC between group 1 and 3 at 3 months. At 7 years no difference in ECC was seen between the groups

In this material, we found a significant difference in central ECC between groups 1 and 3 at 3 months, but at 7 years no difference in ECC was seen between the groups (Table 2, Figs. 1, 2).

**Table 2:** Endothelial cell loss at different intervals

Group^a^	ECL^b^pre-3mo	ECLpre-7y	ECL3mo-7y
1	−125 ± 133	−456 ± 271	−331 ± 321
2	−368 ± 269	−581 ± 280	−213 ± 294
3	−1240 ± 359	−1239 ± 422	1 ± 213
Clear	−246 ± 240	−518 ± 260	−272 ± 284
Not clear	−1240 ± 359	−1239 ± 422	1 ± 213

^a^Increase in central corneal thickness day one after the cataract surgery—group 1: < 5% increase, group 2: 6% to 20% increase, and group 3: ≥ 20% increase. Corneas were defined as “clear” and “not clear” depending on if a specular microscope endothelial photography at day one could be obtained or not

^b^ECL: Endothelial cell loss at different time intervals

## Discussion

To my knowledge, this is the first prospective study of corneal endothelial cell status after phacoemulsification cataract surgery up to 7 years.

The ECL was significantly greater in group 3 at 7 years although the total ECC at 7 years did not differ between the groups. This can somehow appear contradictory, but since there seem to be a tendency for a converging state among the groups, the ECL may as well not differ in a future time point. The statistics, with a wider range in standard deviation in the ECL calculations, may also have contributed to the results.

Observing the ECC at different time points, one can hypothesize that the initial surgical trauma initiates a long-term ECL that is predestined regardless of the early ECL. The greater cell loss in group 3 up to 3 months is compensated by the lack of further loss up to 7 years, whereas the not significant cell loss in group 1 up to 3 months progressed significantly up to 7 years.

The same pattern was seen in the comparison between clear and not clear corneas where the initial cell loss in the not clear group was compensated by no loss between 3 months and 7 years. On the other hand, a greater loss was found in the clear corneas between 3 months and 7 years with no difference in ECC at this time point.

It is stated that corneal endothelial cells cannot regenerate. However, studies have questioned the existing paradigm concerning corneal endothelial wound healing. Evidence has emerged that healthy peripheral cells in the cornea can compensate for the ECL and restore function. Maybe this is a part of the explanation regarding the results [15].

The time point 7 years is a comparably long interval from 3 months. The time for the follow-up was merely chosen since the opportunity and financing came up at that moment. It is likely that the results could be obtained earlier since the cells likely stabilize at an earlier time point. However, an even longer follow-up could be of interest to establish whether there is a complete steady state. The medium age for cataract surgery in Sweden is 74.6 years and the medium life span is 82.55 years, so it can be encouraging that even if there is an initial large cell loss, it can level out and the cornea can stabilize with a lower risk for decompensation[16.]

A current retrospective study found that a 10-year ECL was 20.62 ± 13.63%[17.] They also showed that the overall cell loss was correlated to the degree of early corneal swelling. In our study, the total cell loss was 23.46 ± 13.94%, but unlike the 10-year study we included brunescent cataracts. In our previous study, nucleus colour was the only pre- or intraoperative variable that independently correlated with the central endothelial cell loss. All four lenses with nucleus colour grade 5 were in group 3, and all these eyes had unclear corneas at the first postoperative day. As discussed above, it can somewhat be reassuring that a hard lens leading to an initial substantial corneal oedema and ECL, in the long term, does not seem to have a lesser ECC. The crucial point in these cases is the preoperative ECC and if it is too low, the patients cornea may decompensate permanently after the cataract operation[18–20.] On the other hand, in patients with guttata and a significant postoperative corneal oedema, one can benefit from waiting and observe since the cell loss might not progress further. This is also what is recommended in some literature[20.]

Corneal endothelial cell capacity is not only dependent on the number of endothelial cells but also how the existing cells function. There is no feasible way to check the function of the remaining cells, but the corneal thickness is one way to indirectly evaluate the function. The cell function may partly also be reflected by the changes in HSF, DE and CV. After the surgical trauma, the cells become more variable in shape, but in time they “come to rest” with a more normal function.[6, 21] According to the literature, the morphology parameters will probably stabilize much earlier and did not differ between the groups at 7 years. An equivalent function in the groups can be assumed since there were no differences between the groups in VA and corneal thickness at the 7-year follow-up.

The Orbscan II slit-scan method to measure the corneal thickness was chosen because it was used in the primary study of the three groups, so that the same method was applied. One benefit of this method compared to i.e. ultrasound is that it can be more consistent when comparing different areas of the cornea. The slit-scan method can be less accurate than i.e. ultrasound, Scheimpflug, or OCT imaging, but in this case this method had to be used in order to achieve a correct comparison.

Regarding the sample size, the groups were small but were taken from the first study with three groups of ten patients each. The results should therefore be interpreted with some caution. Nevertheless, to my knowledge, this is the longest prospective follow-up of detailed corneal endothelial cell changes after phacoemulsification cataract surgery.

## Conclusion

The study indicates that regardless of the initial ECL and degree of corneal oedema, the ECC appears to converge towards a long-term steady state.
